# Absence of the axon initial segment in sensory neuron enhances resistance to amyotrophic lateral sclerosis

**DOI:** 10.1093/brain/awaf182

**Published:** 2025-07-07

**Authors:** Nguyen Thu Tra, Sumiko Kiryu-Seo, Haruku Kida, Koji Wakatsuki, Yoshitaka Tashiro, Motosuke Tsutsumi, Mitsutoshi Ataka, Yohei Iguchi, Tomomi Nemoto, Ryosuke Takahashi, Masahisa Katsuno, Hiroshi Kiyama

**Affiliations:** Department of Functional Anatomy and Neuroscience, Nagoya University, Graduate School of Medicine, Nagoya 466-8550, Japan; Department of Functional Anatomy and Neuroscience, Nagoya University, Graduate School of Medicine, Nagoya 466-8550, Japan; Department of Functional Anatomy and Neuroscience, Nagoya University, Graduate School of Medicine, Nagoya 466-8550, Japan; Department of Functional Anatomy and Neuroscience, Nagoya University, Graduate School of Medicine, Nagoya 466-8550, Japan; Department of Neurology, Kyoto University, Graduate School of Medicine, Kyoto 606-8507, Japan; Biophotonics Research Group, Exploratory Research Center on Life and Living Systems, National Institutes of Natural Sciences, Okazaki 444-8787, Japan; Research Division of Biophotonics, National Institute for Physiological Sciences, National Institutes of Natural Sciences, Okazaki 444-8787, Japan; Biophotonics Research Group, Exploratory Research Center on Life and Living Systems, National Institutes of Natural Sciences, Okazaki 444-8787, Japan; Research Division of Biophotonics, National Institute for Physiological Sciences, National Institutes of Natural Sciences, Okazaki 444-8787, Japan; Department of Neurology, Nagoya University, Graduate School of Medicine, Nagoya 466-8550, Japan; Biophotonics Research Group, Exploratory Research Center on Life and Living Systems, National Institutes of Natural Sciences, Okazaki 444-8787, Japan; Research Division of Biophotonics, National Institute for Physiological Sciences, National Institutes of Natural Sciences, Okazaki 444-8787, Japan; Department of Neurology, Kyoto University, Graduate School of Medicine, Kyoto 606-8507, Japan; Research Administration Center, Kyoto University (KURA), Kyoto 606-8507, Japan; Department of Neurology, Nagoya University, Graduate School of Medicine, Nagoya 466-8550, Japan; Department of Functional Anatomy and Neuroscience, Nagoya University, Graduate School of Medicine, Nagoya 466-8550, Japan; Shijonawate Gakuen University, Osaka 547-0001, Japan

**Keywords:** amyotrophic lateral sclerosis, proteostasis, neuronal injury, neurodegeneration, axonal transport, Rpt3 (Pmsc4)

## Abstract

Amyotrophic lateral sclerosis (ALS) is a neurodegenerative disease characterized by the selective loss of motor neurons. Proteasome dysfunction in ALS is considered to cause the accumulation of protein aggregates, which leads to motor neuron degeneration; however, the resilience of motor neurons to ALS pathology might be impaired long before the appearance of protein aggregates. Intriguingly, sensory dorsal root ganglion (DRG) neurons are not susceptible to ALS pathology despite their processes coexisting with axons of motor neurons in the same spinal nerves. Both DRG neurons and motor neurons in ALS model mice express activating transcription factor 3 (ATF3), a well-known marker of nerve injury and disease progression, suggesting that both types of neurons respond to ALS pathology. However, it remains unknown why only DRG neurons are resilient to ALS pathological damage.

To address this issue, we used a nerve injury model in combination with unique injury-induced genetically engineered mice, in which genetic control with an *Atf3* regulatory element enables proteasome ablation and mitochondrial visualization specifically in damaged neurons.

Using the strategy, we found that DRG neurons are resistant to damage in proteasome-deficient conditions, whereas spinal motor neurons degenerate in the same conditions. This might be because DRG neurons lack the typical axon initial segment (AIS), which normally exists in mature neurons and acts as a gate for the selective transport of cargo to axons. The absence of a typical AIS in DRG neurons facilitated increased entry of mitochondria into the axon upon injury, with or without proteasome function. In contrast, damaged motor neurons lacking the proteasome failed to disassemble the AIS, which prevented increased mitochondrial influx into axons and led to energy depletion and degeneration. In the absence of the AIS, DRG neurons in the ALS mouse model are able to deliver sufficient mitochondria into the axon to prevent pathological damage. However, impaired proteasome function in ALS motor neurons results in retention of the AIS gate and failure of mitochondrial transport to axons. This is a possible reason why DRG neurons have greater resilience to ALS pathological damage compared with spinal motor neurons. Collectively, this study opens new directions for the understanding of neurodegenerative diseases at early stages of disturbed protein homeostasis.

## Teaser

Sensory dorsal root ganglion (DRG) neurons are resistant to ALS pathology. Tra *et al.* suggest that this may be due to the absence of the axon initial segment, a structure that restricts cargo transport into the axon. DRG neurons without this structure can increase mitochondrial energy supply into the axon upon ALS damage.

## Introduction

Amyotrophic lateral sclerosis (ALS) is a neurodegenerative disease that is characterized by the progressive loss of motor neurons.^1^ A key hallmark of ALS pathology is disturbed protein homeostasis, which causes the accumulation of protein aggregates and leads to neuronal dysfunction and neurodegeneration.^2^ However, it is likely that neurons are pathologically damaged long before the observable accumulation of protein aggregates. Activating transcription factor 3 (ATF3) is a stress-responsive transcription factor and a well-established marker for nerve injury.^3-5^ ATF3 expression is induced in injured neurons and in pathological neurons damaged by disease or disease-associated gene mutants.^6-9^ ATF3 expression is also induced in motor neurons in presymptomatic stage ALS in response to pathological damage, and the number of ATF3+ motor neurons increases with disease progression.^8^ ATF3 initiates an injury-response transcriptional programme. Therefore, ATF3-expressing ALS motor neurons exhibit numerous stress responses that are similar to those of regenerating motor neurons after axotomy; however, they eventually degenerate. This suggests that ALS motor neurons fail to activate resilience mechanisms against pathological damage prior to the accumulation of protein aggregation.

Other cell types also respond to ALS pathology in mouse models and human patients, but do not degenerate.^10,11^ For instance, the sciatic nerve consists of axons extending from spinal motor neurons and sensory dorsal root ganglion (DRG) neurons, and in sciatic nerves of ALS model mice, hyper-activated macrophages cause the selective degeneration of spinal motor neurons.^12^ However, both motor and DRG neurons in ALS accumulate mutant SOD1, an ALS-associated protein,^13^ suggesting a similar protein homeostatic stress burden. Previous reports suggest that DRG neurons have different sensitivity to disease pathology compared with motor neurons.^11,14-16^ Therefore, sensory DRG neurons might acquire high thresholds against pathological damage in response to disturbed protein homeostasis.

Protein-degradation systems are crucial for cellular protein homeostasis. Of the two major protein-degradation systems, the proteasome system and the autophagy system, earlier signs of dysfunction occur in the proteasome system in ALS pathology.^17,18^ However, it remains unclear how DRG neurons in ALS acquire resilience to pathological damage subsequent to proteasome dysfunction. Previously, we identified a proteasome-dependent damage response in which ankyrin G (AnkG), an organizer of the axon initial segment (AIS), is a crucial target of the proteasome in damaged motor neurons.^19^ The AIS is a specialized compartment located at the beginning of the axon, in which specific membrane proteins, such as channel and adhesion molecules, are concentrated using AnkG as a scaffold. Normally, the AIS functions as a gate for selective cargo transport and as an action potential generator.^20,21^ In response to axon injury, the AIS in motor neurons undergoes proteasome-mediated disassembly, resulting in the loss of the AIS gate function. This, in turn, increases the influx of mitochondria into the axon from the soma, which satisfies the energy demand in the distal part of the axon to prevent motor neuron degeneration. In contrast, ALS motor neurons with proteasome dysfunction fail to activate this mechanism in response to pathological damage.^19^ Given that mitochondria are critical organelles for energy supply in axons, a decrease in their number leads to motor neuron degeneration in ALS patients and model mice.^22,23^ In contrast, ALS sensory DRG neurons escape from ALS pathology, although the existence of the AIS is controversial in DRG neurons.^24-26^ It is currently unknown whether ALS DRG neurons properly regulate the gate function of the AIS in response to pathological damage when proteasome function is impaired or whether other compensatory functions are activated.

To address this issue, we used *Atf3*:bacterial artificial chromosome transgenic (BAC Tg) mice, in which the induction of Cre recombinase and the labelling of mitochondria by GFP are under the control of an *Atf3* gene regulatory element and occur only in injured or diseased neurons.^19,27^ The difference in damage response under proteasome deficiency between sensory DRG neurons and spinal motor neurons can be detected in these mice. The sensory DRG neurons do not have a typical AIS structure, allowing the transport of mitochondria into damaged axons even in proteasome-deficient ALS conditions. This is a possible reason why DRG neurons have greater resilience to ALS pathological damage compared with spinal motor neurons.

## Materials and methods

### Animals

All protocols were conducted according to the Guidelines for the Care and Use of Laboratory Animals of Nagoya University Graduate School of Medicine and were approved by the Nagoya University Institutional Animal Care and Use Committee. All experiments follow the 3R principle (replacement, refinement and reduction) and the ARRIVE guidelines.

SOD1^G93A^ mice [B6SJL-Tg(SOD1*G93A)1Gur/J, stock number 002726, B6SJL background] and *Thy1*-Mito mice [B6.Cg-Tg(Thy1-CFP/COX8A)S2Lich/J, stock number 007967] were obtained from the Jackson Laboratory and were maintained according to Jackson Laboratory's protocol. The genotyping protocols for *Thy1*-Mito and SOD1^G93A^ mice are available through the Jackson Laboratory.

We previously generated *Atf3*:BAC and *Atf3*:BAC2 Tg mice.^19,27^ To generate injury-induced proteasome-deficient mice (*Rpt3* CKO), *Atf3*:BAC2 Tg mice were crossed with *Rpt3^flox/flox^* mice (provided by Drs Y. Tashiro and R. Takahashi, Kyoto University).^18,19^ Tail lysates from the offspring of this cross were genotyped by PCR using specific primer pairs: *Atf3*:BAC2-F 5′-CAATAAGATGGAGTACAACTACAACGC-3′ and *Atf3*:BAC2-R 5′-GACTCTTTCCACAACTATCCAACTCAC-3′, and *Rpt3*-F 5′-TGAGCTGTGTATCAAGGTCC-3′ and *Rpt3*-R 5′-TAGAAGCTGCCTAAGGCACA-3′. To detect deletion of the *Rpt3* gene, *Rpt3*-delta 5′-TGCAATCCCTTGTCAGGAGA-3′ was used. Homozygous *Rpt3^flox/flox^* mice were maintained by crossing with *Rpt3^flox/flox^* mice, which had not been crossed with Cre driver mice to avoid unexpected germline recombination as described previously.^19^

We also previously generated *Atf3*:BAC;SOD1^G93A^ (*Atf3*;SOD1) mice.^19^ Briefly, SOD1^G93A^ mice were crossed with *Atf3*:BAC Tg mice on a B6SJL background to generate *Atf3*;SOD1 mice.

All mice were matched for age and sex. Experiments were performed on both sexes of *Atf3*:BAC2 Tg, *Rpt3* CKO and *Thy1*-Mito mice. For the ALS mouse model, male SOD1^G93A^ mice, male *Atf3*;SOD1 mice and male littermates with or without the *Atf3*:BAC transgene were recruited for experiments. The end-stage of SOD1^G93A^ mice was defined as a weight loss of 15% and/or severe paralysis in both hindlimbs together with an inability to perform a righting test within 20 s. These criteria are frequently used in ALS mouse model assessment.

### Surgical procedures

Mice 10–12 weeks of age were anaesthetized with isoflurane. The right sciatic nerve was exposed and transected with a pair of scissors at the mid-thigh level, immediately before the bifurcation of the sciatic nerve into tibial and common peroneal nerves. The incision was closed with a single 2.0 cotton silk suture.

### Histological sample preparation and immunohistochemistry

Mice were perfused transcardially with Zamboni solution [2% paraformaldehyde, 0.2% picric acid in 0.2 M phosphate buffer; or for AnkG staining, 1% paraformaldehyde, 0.2% picric acid in 0.2 M phosphate buffer]. Spinal cord from L4–L5 and DRGs from L4 and L5 with an attached dorsal root, ventral root and spinal nerves were collected and post-fixed in Zamboni solution overnight at 4°C, then kept in 30% sucrose solution until the tissue sank. Samples were embedded in O.C.T. compound, then serially sectioned on a cryostat at 10 μm thickness for DRG samples and at 20 μm for spinal cord samples and mounted on slide glasses.

For free-floating immunohistochemistry, DRG and spinal cord samples were sectioned at 50 μm thickness. Sections were then washed in 0.01 M PBS, blocked in 0.01 M PBS containing 1% bovine serum albumin and 0.3% Triton X-100, then incubated at 4°C overnight with primary antibodies (Supplementary Table 1). Incubation with anti-TrkA, anti-TrkB, anti-TrkC and anti-tyrosine hydroxylase (TH) antibodies was performed at room temperature. Sections were then washed in 0.01 M PBS and incubated with conjugated secondary antibodies and 4′,6-diamidino-2-phenylindole (DAPI) for nuclear staining.

### Thionine staining

DRGs from L4–L6 and the relevant spinal cord segment were dissected for all cell counting investigations. DRG sections 10-μm-thick and spinal cord sections 20-μm-thick were washed with 0.01 M PBS for 30 min, stained with thionine for 3 min at room temperature, then washed with distilled water. The sections were then dehydrated by two 10 min incubations in each of 70%, 80%, 90% and 100% alcohol solutions and were then cleared by three 10 min incubations in xylene. The thionine-stained sections were observed using a BZ-X800 microscope (Keyence) with light filter and a 10× objective.

### Cell counting

Survival rates of spinal motor and DRG neurons were investigated at four time points of model ALS progression [young age (7 and 8 weeks old), presymptomatic stage (10–12 weeks old), symptomatic stage (13–17 weeks old) and end-stage (18–22 weeks old)] and were compared with wild-type littermate controls. The survival rates of spinal motor and DRG neurons on the injured side of SOD1^G93A^ mice were evaluated at 28 days after nerve transection and were compared with the non-injured side and control mice. To avoid double counting, every eighth section was counted from L4 and L5 of each mouse. Counted neurons were defined as those that contained a clear nucleus and definite cytoplasm. Twenty to thirty DRG sections and 8–10 spinal cord sections were counted per mouse. The DRG area in sections was also calculated, and the survival rate of DRG neurons was defined as the density of neurons per millimetre squared.^28^ The survival rate of spinal motor neurons was quantified as the number of spinal motor neurons per section. Only motor neurons with a large soma, a clear nucleus with intact nuclear membrane and at least one large clump of nucleolar material were counted.^29^ All cell counting was performed using Fiji software (NIH, Bethesda, MD, USA).

ATF3+ DRG neurons and ATF3+ spinal motor neurons were counted in every eighth section (to avoid counting the same cell twice). As described above, 20–30 DRG sections and 8–10 spinal cord sections were counted per mouse. ATF3-expressing neurons were quantified as the number of ATF3+ neurons per DRG and spinal cord section. The efficiency of GFP expression in injured DRG neurons of *Atf3*:BAC2 Tg mice was determined as the percentage of GFP+ neurons among ATF3+ neurons using Fiji software (NIH).

### Whole-mount observations of axonal mitochondria

Fluorescently labelled mitochondria were visualized in intact, injured and diseased nerves. L4–L6 DRGs with 4 mm of dorsal root, ventral root and spinal nerve attached were dissected from mice after perfusion with Zamboni solution. The central branch of the dorsal root and ventral root were used for imaging. Consecutive *z*-stack series throughout the whole thickness of the nerve roots were acquired using a confocal laser scanning microscope with a 60× oil-immersion objective (Nikon TiE-A1R) or a 60× water-immersion objective (Olympus FV10i). The quantification of GFP-labelled mitochondria in nerve roots is presented as the number of mitochondria per 50 µm^2^ counted in a single axon.^19^ Five to eight nerves from each mouse were used for counting. Counting was performed using Fiji software (NIH).

### Image analysis

Images were acquired using a BZ-X800 inverted fluorescence microscope (Keyence) with a 10×, 20× or 40× objective, or with an FV10i confocal laser scanning microscope (Olympus) using a 10× objective or a 60× water-immersion objective (NA 1.2). All image analyses were performed using Fiji software (NIH). The GFP+ mitochondria in the AIS or predicted AIS region were counted and are presented as the number in the AIS region 10 µm in length. Ten to twenty neurons from each mouse were examined. The length of the AIS stained by AnkG was measured using *z*-stacked images. For AIS length, 20–30 neurons were examined in each mouse, and for AIS number, three to six areas.

For high-resolution 3D imaging of DRG neurons, 50-μm-thick tissue sections were mounted on glass slides using ProLong Glass Antifade Mountant (Thermo Fisher Scientific). Images were captured using a TCS SP8 laser confocal microscope (Leica Microsystems) equipped with an HC PL APO CS2 100×/1.40 oil-immersion objective (Leica Microsystems). Alexa Fluor 488 and Alexa Fluor 594 dyes were excited at 488 and 594 nm, respectively, with a supercontinuum white light laser. The emitted fluorescence was detected sequentially using HyD-SMD detectors through a spectral separation module (505–550 nm for Alexa Fluor 488 and 605–700 nm for Alexa Fluor 594). The pinhole size was set at 0.40 Airy units (60.7 μm). The pixel size and *z*-step were set at 45 and 182 nm, respectively. The acquired images were processed using the LIGHTNING deconvolution algorithm in LAS-X software (Leica Microsystems). 3D reconstructions of the images were generated using NIS-Elements software (Nikon).

### Reinnervation

Footpad tissue sections 30-μm-thick were stained for the pan-neuronal marker, TUJ1. To assess epidermal and dermal reinnervation, the number of intraepidermal nerve fibres indicated by TUJ1 staining was measured. To estimate the density of reinnervation in the epidermis, images of randomly selected areas of footpad skin were taken, and the number of TUJ1+ nerve fibres in the epidermis was counted. The dermis–epidermis borderline was traced and its length measured using Fiji software. At least 10 areas were analysed for each mouse. The average number of TUJ1+ fibres per unit length of the borderline was determined.

### Statistical analysis

All reported results in figures show individual data-points and/or the mean ± standard error of the mean (SEM) error bars. Data were analysed using Student's unpaired *t*-test for two-group comparison and one-way ANOVA followed by Tukey's *post hoc* test for comparison of multiple groups. The significance level was set at ≤0.05. All graphs were generated and statistical analyses performed using Graphpad Prism software v.9.3.1. Details of the statistical analysis are provided in each figure legend.

## Results

### Sensory DRG neurons survive pathological damage in SOD1^G93A^ mice

**Figure 1:**
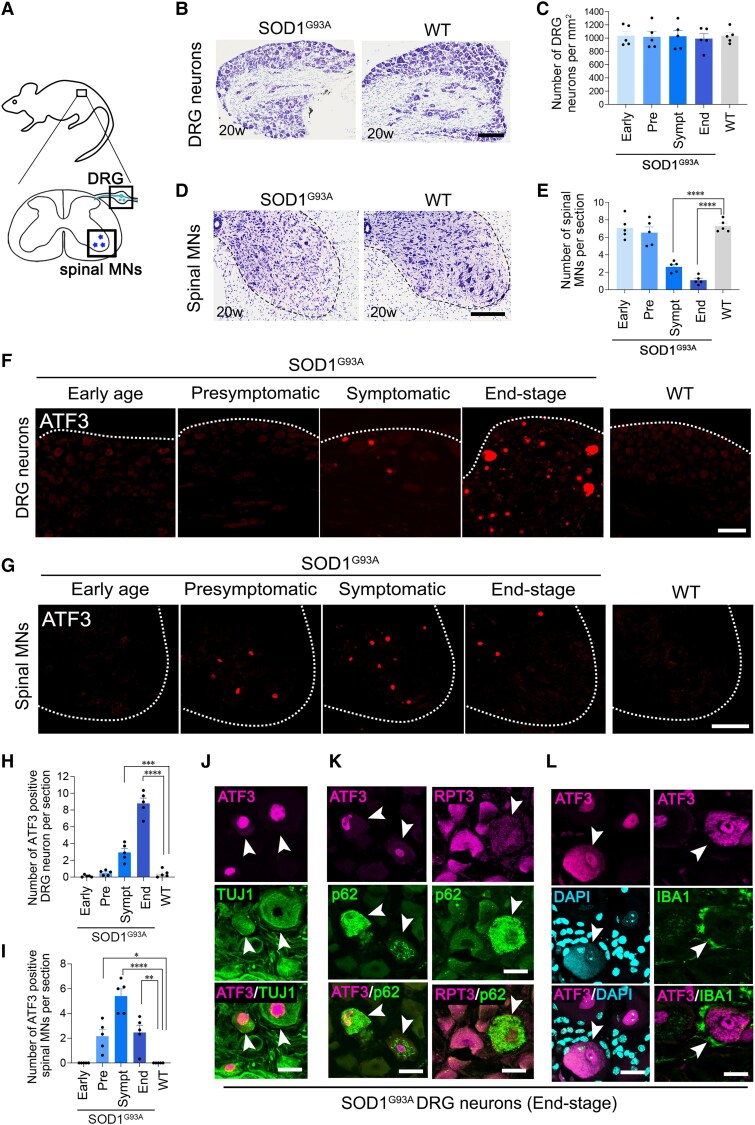
**Sensory DRG neurons show resilience against pathological damage in SOD1^G93A^ ALS mice.** (**A**) Schematic illustration showing DRG and spinal cord in the boxes. (**B**) Representative thionine-stained DRG neurons of SOD1^G93A^ mouse and WT mouse at 20 weeks old (disease end-stage in SOD1^G93A^ mice). (**C**) Quantification of the number of DRG neurons per millimetre squared at different disease stages. (**D**) Representative thionine-stained lumbar spinal motor neurons of SOD1^G93A^ mouse and WT mouse. Dashed lines outline the boundary of grey matter in the spinal cord. (**E**) Quantification of the number of spinal motor neurons per section. (**F** and **G**) Immunostaining of DRGs and spinal cord for of ATF3 in SOD1^G93A^ mice at different disease stages and 20-week-old control mouse (WT). Dashed lines reveal the edge of DRG and the boundary of grey matter in the spinal cord. (**H** and **I**) Quantification analyses showing the number of ATF3+ cells per section in DRG (**H**) and spinal cord (**I**) in SOD1^G93A^ mice and WT mice. (**J**–**L**) Immunostaining of ATF3 and TUJ1 (**J**), ATF3 and p62 or RPT3 and p62 (**K**), and ATF3 and DAPI or ATF3 and IBA1 (**L**) in DRGs of a SOD1^G93A^ mouse at end-stage. Filled arrowheads indicate the co-localization of ATF3 with each marker in **J**–**L** and that of RPT3 with p62 in **K**. Data are expressed as the mean ± standard error of the mean. *P = 0.0182, **P = 0.0063, ***P = 0.0008 and ****P < 0.0001, determined by one-way ANOVA followed by Tukey’s *post hoc* analysis. *n* = 5 mice for each group. Scale bars = 100 µm in **B**, **D**, **F**, **G**; 10 µm in **J**–**L**. ALS = amyotrophic lateral sclerosis; DRG = dorsal root ganglion; MNs = motor neurons; WT = wild-type.

We observed thionine-stained sections of DRG neurons and spinal motor neurons from SOD1^G93A^ and wild-type littermate control mice (Fig. 1A). In end-stage (20-week-old) SOD1^G93A^ mice, almost no DRG neurons showed apparent degenerative features (Fig. 1B). We counted the number of DRG neurons at four different stages of disease progression [early stage (7–8 weeks old), presymptomatic (10–12 weeks old), symptomatic (13–17 weeks old) and end-stage (18–22 weeks old)] to evaluate cell survival. There was no significant difference in survival rate of DRG neurons between SOD1^G93A^ and wild-type mice throughout the disease progression (Fig. 1C). In contrast, the survival rate of spinal motor neurons in SOD1^G93A^ mice declined progressively with disease progression, which is consistent with previous studies^30,31^ (Fig. 1D and E). These observations prompted us to examine how DRG neurons are resistant to ALS-associated pathology.

It is widely accepted that pathological damage in ALS motor neurons activates many stress responses. We therefore next examined whether DRG neurons in SOD1^G93A^ mice respond to pathological damage. Cultured DRG neurons from ALS mice express ATF3^32,33^; therefore, we evaluated the levels of ATF3 in DRGs by immunohistochemistry, because ATF3 is a stress-responsive transcription factor and a well-known marker for nerve injury and pathological damage.^34^ As shown in Fig. 1F, DRGs in SOD1^G93A^ mice express ATF3 at the symptomatic stage, and the number of DRGs expressing ATF3 was increased at the end-stage (Fig. 1F and H). Wild-type mice did not show any apparent expression of ATF3 in DRGs. In contrast, in SOD1^G93A^ spinal motor neurons, the expression of ATF3 was induced at the presymptomatic stage, reached a peak level at the symptomatic stage, then declined at the end-stage because of motor neuron death (Fig. 1G and I). The expression of ATF3 in DRGs of SOD1^G93A^ mice was delayed in comparison to that in spinal motor neurons of SOD1^G93A^ mice. The ATF3-expressing cells in DRGs co-localized the neuronal marker, TUJ1, indicating that these cells are neurons (Fig. 1J). Moreover, the ATF3+ DRG neurons of SOD1^G93A^ mice accumulated p62, which is a marker of autophagy activation (Fig. 1K). Notably, p62+ neurons have reduced abundance of the proteasome subunit, RPT3,^35^ indicating that the ATF3+ DRG neurons increased activation of compensatory autophagy because of proteasome insufficiency. To identify the ATF3+ DRG neuron subtype in SOD1^G93A^ mice at the end-stage, we performed immunostaining using TrkA, TrkB, TrkC and TH antibodies (Supplementary Fig. 1). However, the ATF3+ DRG neurons showed no preference for these markers. We did find a limited number of DRG neurons with cytoplasmic ATF3 at the end-stage (Fig. 1L, arrowhead). These DRG neurons were highly damaged or degenerating, because they exhibited DAPI staining in the cytoplasm, indicating DNA leakage, and were surrounded by IBA1+ macrophages. Together, these data indicate that most DRG neurons survive until the later stage of ALS, although they accrue pathological damage. This is probably because damaged DRG neurons have a mechanism that provides resilience in the presence of proteasome dysfunction.

### ALS pathology does not affect the resilience of DRG neurons to damage

**Figure 2:**
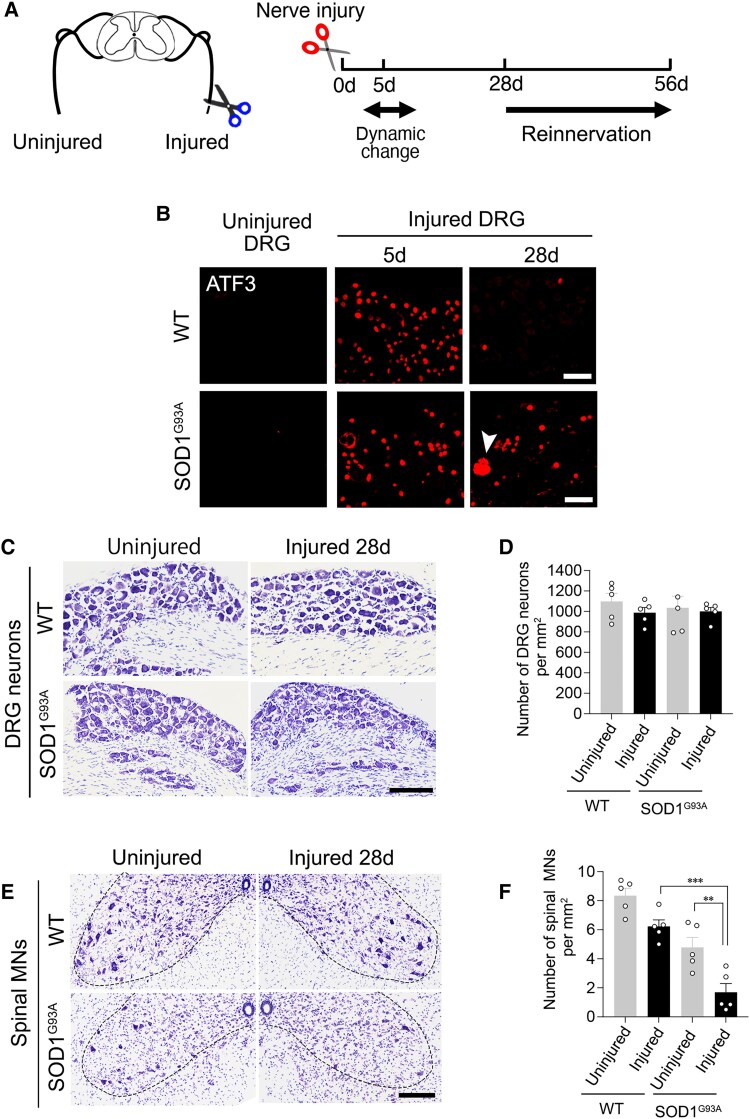
**DRG neurons of SOD1^G93A^ ALS mice survive after sciatic nerve injury.** (**A**) Schematic diagram of sciatic nerve injury model. (**B**) The expression of ATF3 in DRG neurons of WT and SOD1^G93A^ mice before and after sciatic nerve injury. Arrowhead shows DRG neuron with cytoplasmic ATF3. (**C**) Thionine-stained DRGs in control and injured sides of WT and SOD1^G93A^ mice at 28 days after axotomy. (**D**) The number of control and injured DRG neurons in WT and SOD1^G93A^ mice at 28 days after sciatic nerve injury. (**E**) Thionine-stained spinal motor neurons in WT and SOD1^G93A^ mice at 28 days after axotomy. (**F**) The number of control and injured spinal motor neurons in WT and SOD1^G93A^ mice at 28 days after nerve injury. Scale bars = 50 µm in **B**; 100 µm in **C** and **E**. Data are shown as the mean ± standard error of the mean, **P = 0.039 and ***P = 0.0001, determined by one-way ANOVA followed by Tukey’s *post hoc* analysis, *n* = 5 mice. ALS = amyotrophic lateral sclerosis; DRG = dorsal root ganglion; WT = wild-type.

We asked whether ATF3-expressing DRG neurons in SOD1^G93A^ mice degenerate because of the gradual accumulation of damage from severe stress or whether they are resistant to damage and survive. SOD1^G93A^ mice have a limited lifespan; therefore, it was difficult to answer this question using this disease model. Instead, we used a sciatic nerve injury model, in which all DRG neurons express high levels of ATF3 (Fig. 2A).^5^ We transected the sciatic nerve of 10- to 12-week-old SOD1^G93A^ and wild-type mice. The expression of ATF3 was induced dramatically in DRG neurons of wild-type and SOD1^G93A^ mice at 5 days after sciatic nerve injury (Fig. 2B). At this time point, injured neurons induce numerous cellular responses (Fig. 2A).^36-38^ The levels of ATF3 in most wild-type but not SOD1^G93A^ mouse neurons returned to control levels at 28 days after injury (Fig. 2B). A small population of DRG neurons in SOD1^G93A^ mice at 28 days after injury showed cytoplasmic ATF3 (Fig. 2B, arrowhead), in a similar manner to that in end-stage SOD1^G93A^ DRG neurons (Fig. 1F and L). We next examined neuronal fate at 28 days after injury (Fig. 2C–F). We chose this time point because of the limited lifespan of SOD1^G93A^ mice and because injured nerves of wild-type mice start to reinnervate from this time point. The numbers of DRG neurons in uninjured and injured sides were not significantly different between wild-type and SOD1^G93A^ mice. In contrast, the spinal motor neurons of SOD1^G93A^ mice were degenerated at 28 days after sciatic nerve injury (Fig. 2E and F).^39^ These findings indicate that SOD1^G93A^ DRG neurons are capable of resisting damage induced by ALS pathogenesis.

### DRG neurons are resistant to injury-induced proteasome deficiency

**Figure 3:**
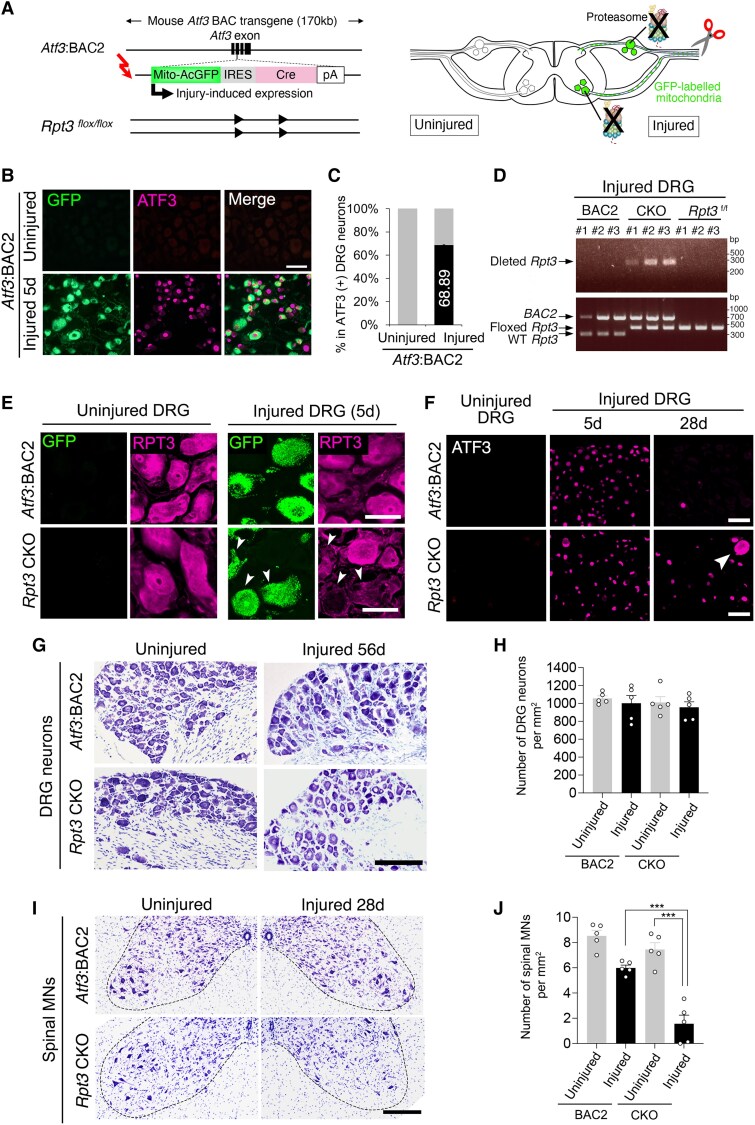
**Injury-induced proteasome-deficient DRG neurons survive after nerve injury.** (**A**) Schematic diagram for deletion of the proteasome subunit *Rpt3*, in injured DRG and spinal motor neurons. *Left*: Black boxes indicate exons in *Atf3*:BAC2 transgene. The Mito-AcGFP IRES Cre cassette was inserted at the site of the initiation codon of *Atf3*. Arrowheads in *Rpt3* gene denote loxP sites. *Right*: *Rpt3* deletion and mitochondrial labelling occur at the same time in injured neurons. (**B**) The expression of GFP and ATF3 in DRGs before and after sciatic nerve injury of *Atf3*:BAC2 Tg mouse. (**C**) Graph showing the percentage of GFP+ or GFP− DRG neurons in ATF3+ neurons on uninjured and injured sides at 5 days after injury of *Atf3*:BAC2 Tg mouse (*n* = 5 mice). (**D**) Genotyping results to detect the deleted *Rpt3* gene (*top*) and to identify the mouse line (*bottom*) using the genome samples extracted from injured DRG neurons of *Atf3*:BAC Tg, *Rpt3* CKO and *Rpt3*^f/f^ mice. Full-length gels are shown in Supplementary Fig. 2. (**E**) The expression of GFP and RPT3 in DRG neurons in *Atf3*:BAC2 Tg and *Rpt3* CKO mice before and after sciatic nerve injury. The arrowheads indicate GFP-expressing neurons with decreased expression of RPT3. (**F**) The time-dependent expression of ATF3 in DRG neurons of *Atf3*:BAC2 Tg and *Rpt3* CKO mice after sciatic nerve injury. Arrowhead shows DRG neuron with cytoplasmic ATF3. (**G**) Thionine staining of DRGs in *Atf3*:BAC2 Tg and *Rpt3* CKO mice at 56 days after sciatic nerve injury. (**H**) The number of uninjured and injured DRG neurons per micrometre squared section in *Atf3*:BAC2 Tg and *Rpt3* CKO mice at 56 days after sciatic nerve injury. (**I**) Thionine staining of spinal motor neurons in *Atf3*:BAC2 Tg and *Rpt3* CKO mice at 28 days after sciatic nerve injury. (**J**) The number of uninjured and injured spinal motor neurons per section in *Atf3*:BAC2 Tg and *Rpt3* CKO mice at 28 days after nerve injury. Scale bars = 50 µm in **B** and **E**; 30 µm in **D**; 100 µm in **F** and **H**. Data are shown as the mean ± standard error of the mean. ****P < 0.0001, determined by one-way ANOVA followed by Tukey’s *post hoc* analysis, *n* = 5 mice. CKO = conditional knockout; DRG = dorsal root ganglion; GFP = green fluorescent protein; WT = wild-type.

The next question we addressed was why damaged DRG neurons have a high threshold against further deterioration in ALS pathogenesis. Proteasome dysfunction is an early hallmark of ALS disease,^17,18^ and damaged ALS spinal motor neurons lack a proteasome-mediated stress resilience mechanism, leading to degeneration.^19^ We hypothesized that damaged DRG neurons are resistant to proteasome deficiency. To address this point, we generated injury-induced proteasome-deficient mice by crossing *Atf3*:BAC2 Tg mice with *Rpt3^flox/flox^* mice, as described previously^19^ (*Rpt3* is also known as *Pmsc4*) (Fig. 3A). RPT3 is a critical regulatory subunit of the 26S proteasome. Conventional *Rpt3* knockout (KO) mice die at an early embryonic stage because the proteasome is essential for cellular integrity.^40^ Our unique *Rpt3* conditional knockout (CKO) mouse enables the proteasome to be deleted only in injured neurons, without affecting development and normal neuronal function. The *Atf3*:BAC2 Tg mice were designed to induce Cre recombinase and to label mitochondria with GFP only in injured neurons, via the control of an *Atf3* gene element within a BAC.^19^ The *Atf3*:BAC2 Tg mice do not express exogenous ATF3, and this does not affect the expression of endogenous ATF3.

Figure 3B shows that *Atf3*:BAC2 Tg mice expressed GFP in DRG neurons after nerve injury. Almost all the GFP-expressing DRG neurons in which a nucleus was discerned expressed ATF3, and 68.89% of ATF3+ DRG neurons expressed GFP (Fig. 3C). *Rpt3* was deleted successfully in injured DRGs of *Rpt3* CKO mice but not in either *Atf3*:BAC2 Tg or *Rpt3* flox mice (Fig. 3D and Supplementary Fig. 2). Furthermore, RPT3 protein was deleted successfully only in GFP-expressing injured DRG neurons of *Rpt3* CKO mice, whereas it was intact in both uninjured and injured DRG neurons of *Atf3*:BAC2 mice (Fig. 3E). Marked ATF3 expression was observed in injured DRG neurons of both *Atf3*:BAC2 Tg mice and *Rpt3* CKO mice at 5 days after sciatic nerve injury (Fig. 3F). *Rpt3*-deficient injured DRG neurons maintained the expression of ATF3 at 28 days after injury, when nerve reinnervation started to occur. At this time point, a small population of DRG neurons in *Rpt3* CKO mice showed cytoplasmic ATF3 in a similar manner to the ALS DRG neurons (Fig. 1F and L) and the ALS DRG neurons after injury (Fig. 2B). We next examined the survival rate of DRG and spinal motor neurons after sciatic nerve injury using *Atf3*:BAC2 Tg mice and *Rpt3* CKO mice. Surprisingly, injury-induced proteasome-deficient DRG neurons in *Rpt3* CKO mice did not show any significant difference in survival rate compared with those in *Atf3*:BAC2 Tg mice at 56 days after injury, in which nerve reinnervation was almost complete (Fig. 3G and H). Meanwhile, the number of injury-induced proteasome-ablated spinal motor neurons was dramatically reduced, consistent with previous studies (Fig. 3I and J).^19^

### Mitochondrial entry into axons is increased in injured DRG neurons that lack the proteasome

**Figure 4:**
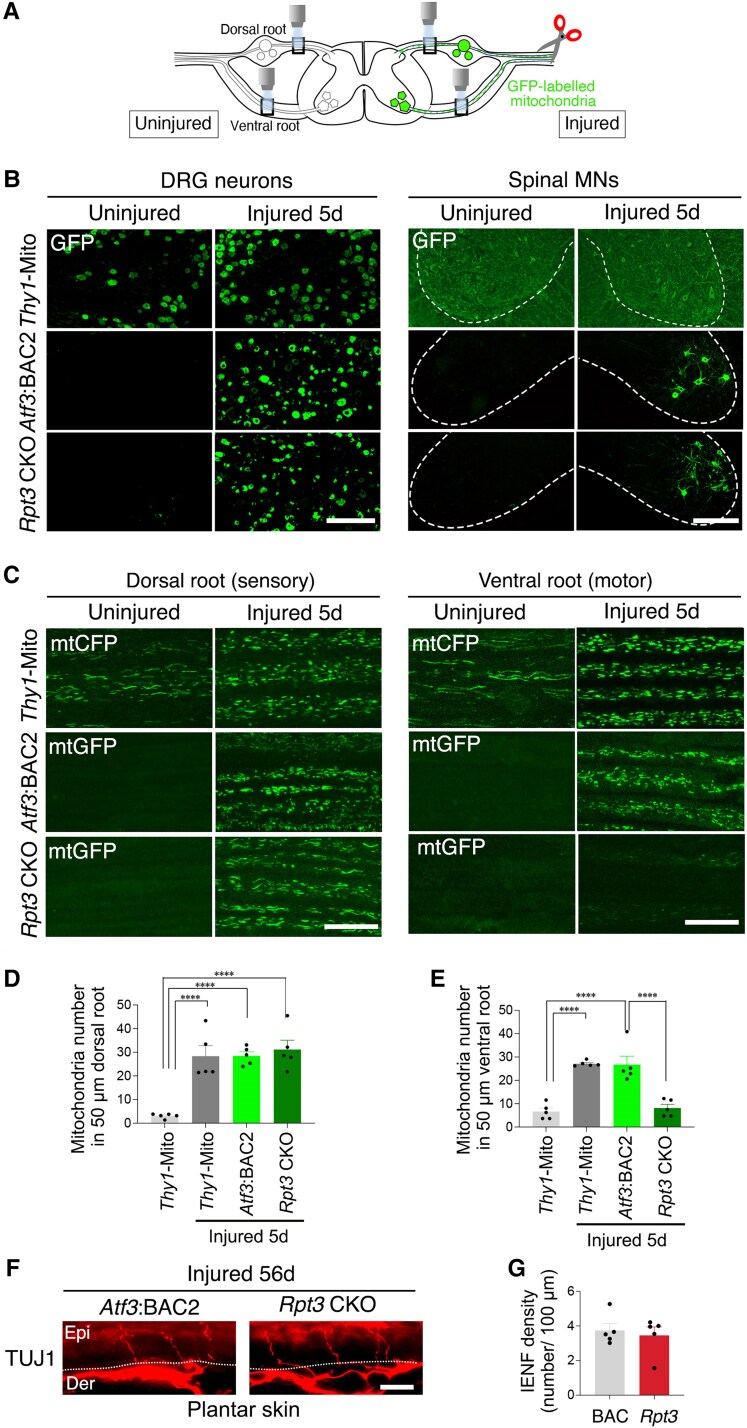
**Injured sensory nerves, but not injured motor nerves, distribute abundant mitochondria under the proteasome deficiency.** (**A**) Schematic diagram of experiment. Mitochondria in the boxed areas of whole nerves were observed under the microscope. (**B**) The immunostaining using GFP antibody for DRG neurons and spinal motor neurons of *Thy1*-Mito, *Atf3*:BAC2 and *Rpt3* CKO mice before and after injury. (**C**) Direct observation of GFP- or CFP-labelled mitochondria (mtGFP or mtCFP) in whole-mount sensory (dorsal root) and motor (ventral root) nerves of *Thy1*-Mito, *Atf3*:BAC2 and *Rpt3* CKO mice before and at 5 days after sciatic nerve injury. (**D** and **E**) The graph shows the number of axonal mitochondria per 50 µm of sensory dorsal root (**D**) and motor ventral root (**E**). (**F**) Re-innervation of TUJ1+ nerve ending in plantar skin of *Atf3*:BAC2 Tg and *Rpt3* CKO mice at 56 days after sciatic nerve injury. (**G**) The graph shows the number of intraepidermal nerve fibres (IENF) per 100 µm length. Scale bars = 100 µm in **B**; 20 µm in **C** and **F**. Data are shown as the mean ± standard error of the mean. ****P < 0.0001, determined by one-way ANOVA followed by Tukey’s *post hoc* analysis, *n* = 5 mice per group. CKO = conditional knockout; DRG = dorsal root ganglion; GFP = green fluorescent protein; MNs = motor neurons; WT = wild-type.

We examined the physiological status of injured DRG neurons further. Mitochondrial behaviour is a good indicator of the physiological status of neurons in normal, traumatic and pathological conditions. Healthy mitochondria are delivered from the soma to the axon tip to supply sufficient energy to maintain nerve integrity; therefore, the transport of mitochondria is a key factor in nerve cell homeostasis. Our mouse system allowed us to visualize GFP-labelled mitochondria specifically in injured neurons and axons (Fig. 4A and B). We observed GFP-labelled mitochondria in whole motor and sensory nerves from *Atf3*:BAC2 Tg mice and *Rpt3* CKO mice, without any bias from sectioning or immunostaining (Fig. 4A and C). We discriminated sensory and motor axons by observing the central branch of dorsal and ventral nerve roots, respectively. *Thy1*-Mito mice, in which mitochondria in neurons are labelled under the control of the *Thy1* promoter, showed that the number of mitochondria was increased in both sensory and motor axons at 5 days after sciatic nerve injury (Fig. 4C). Usually, injured neurons show a dramatic change in cell responses at this time point (Fig. 2A). Consistent with this, the number of mitochondria was increased in injured sensory and motor axons of *Atf*3:BAC2 Tg mice. Intriguingly, the number of mitochondria in the *Rpt3*-deficient injured sensory nerve in the central part of the dorsal root was similar to that in *Atf*3:BAC2 Tg mice, whereas *Rpt3*-deficient injured motor axons in ventral roots showed far fewer mitochondria compared with uninjured axons and injured sensory axons of both mice (Fig. 4C–E). These findings demonstrate that injured sensory DRG neurons maintain the ability to increase the delivery of mitochondria to the axon despite proteasome deficiency, whereas injured motor neurons do not. We then investigated whether proteasome deficiency in injured DRG neurons affects axonal regeneration. *Rpt3* CKO mice showed similar skin reinnervation in the hairless interdigital area of the hindpaw to that in *Atf3*:BAC2 Tg mice at 56 days after nerve injury (Fig. 4F and G).

### Despite proteasome deficiency, injured sensory nerves without an AIS are protected from energy failure

**Figure 5:**
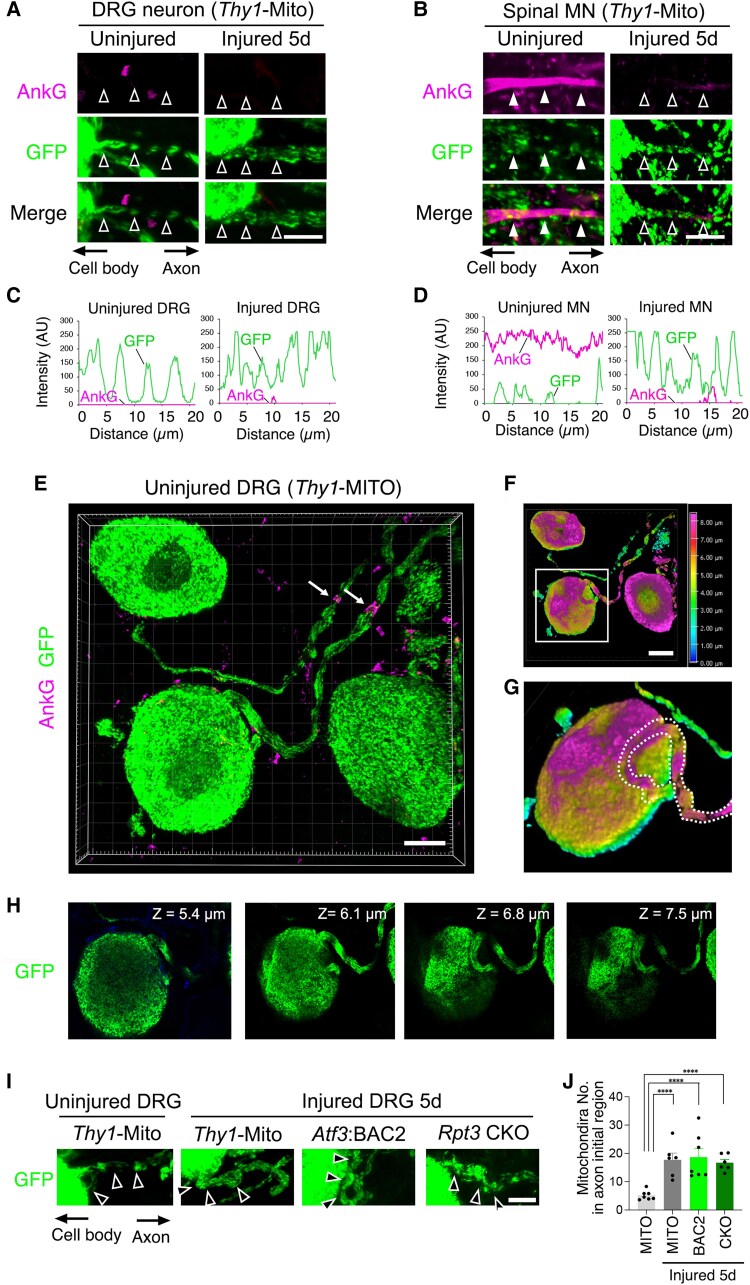
**Sensory DRG neurons have no typical AnkG+ AIS.** (**A** and **B**) The expression of AnkG and GFP in the initial part of the axon in a DRG neuron (**A**) and a spinal motor neuron (**B**) of a *Thy1*-Mito mouse before and after sciatic nerve injury. Open arrowheads show the constitutive existence of GFP-labelled mitochondria in the beginning of the axon, and filled arrowheads show few mitochondria in the AnkG+ AIS region. (**C** and **D**) Graphs show the GFP and AnkG fluorescent intensity scans corresponding to **A** and **B**, respectively, over a 20 μm line running from the soma to the axon. (**E**) 3D images of uninjured DRG neurons in *Thy1*-Mito mouse stained by GFP and AnkG. Arrows indicate the node of Ranvier. (**F**) Pseudo-colour of 3D images in **E** classified by the *z*-direction. White boxed area was magnified in **G**. (**G**) Dotted lines trace the beginning of the axon. (**H**) Serial section in **E** between 5.4 and 7.5 µm of depth. Each section shows the GFP-labelled mitochondria in the initial part of the axon. (**I**) GFP-labelled mitochondria in AIS region of uninjured DRG of *Thy1*-Mito mouse and injured DRGs of *Thy1*-Mito, *Atf3*:BAC2 and *Rpt3* CKO mice. Open arrowheads indicate the beginning of the axon. (**J**) Graph shows the number of mitochondria in the axon initial region. Scale bars = 10 µm in **A**, **B**, **E** and **I**; 25 µm in **F**. Data are shown as the mean ± standard error of the mean. **P < 0.005 and ***P = 0.0005, determined by one-way ANOVA followed by Tukey’s *post hoc* analysis, *n* = 5 mice per group. AIS = axon initial segment; CKO = conditional knockout; DRG = dorsal root ganglion; GFP = green fluorescent protein; MN = motor neuron; WT = wild-type.

Recently, we found that the AIS, which is located at the beginning of the axon, can be disassembled temporarily to increase the entry of mitochondria into the axon upon motor nerve injury.^19^ We therefore examined the morphological dynamics of the AIS and mitochondrial distribution before and after injury, using *Thy1*-Mito mice, in which mitochondria in most neurons are labelled. AnkG is a master organizer of AIS assembly; therefore, we stained for AnkG and GFP in DRG neurons and spinal motor neurons before and after injury (Fig. 5A–D). We did not detect any AnkG staining at the beginning of the axon in uninjured or injured DRG neurons (Fig. 5A and C). However, uninjured spinal motor neurons clearly possessed an AnkG+ AIS, which was dismantled after injury (Fig. 5B and D). The staining for other AIS markers, such as Neurofascin 186 (NF 186) and Na_v_1.6, was also negative at the beginning of the axon in uninjured DRG neurons, in contrast to uninjured spinal motor neurons (Supplementary Fig. 3). GFP-labelled mitochondria were evenly distributed in the proximal region of DRG axons before and after injury. In contrast, mitochondria were absent in the AIS of uninjured spinal motor neurons but were abundant in the AIS region following AIS disappearance after injury. These observations in motor neurons are consistent with our previous findings of mutually exclusive distribution of AIS and mitochondria.^19,41^ Recently, Nascimento *et al*.^25^ reported the existence of the AIS in DRG neurons *in vivo*. Therefore, we carefully examined the AnkG+ signals and the mitochondrial appearance at the beginning of the axon in uninjured DRG neurons using high-resolution of 3D imaging. AnkG positivity was observed in the node of Ranvier, which has similar protein components to the AIS, but not in the beginning of the axon of uninjured DRG neurons (Fig. 5E). Fluorescently labelled mitochondria appeared at the beginning of the axon in uninjured DRG neurons but not in uninjured spinal motor neurons. When the thickness of the 3D image was assigned a different colour, we found a snake-like structure in the beginning of the axon of uninjured DRG neurons (Fig. 5F and G). Mitochondria constantly occupied this region (Fig. 5H). As shown in Fig. 5I and J, injured DRG neurons with or without proteasomes had increased numbers of axonal mitochondria. These findings indicate that injured DRG neurons do not need to dismantle the AIS to increase the number of mitochondria in the damaged axon.

### Absence of the AIS is a possible reason why DRG neurons are resistant to ALS pathology

**Figure 6:**
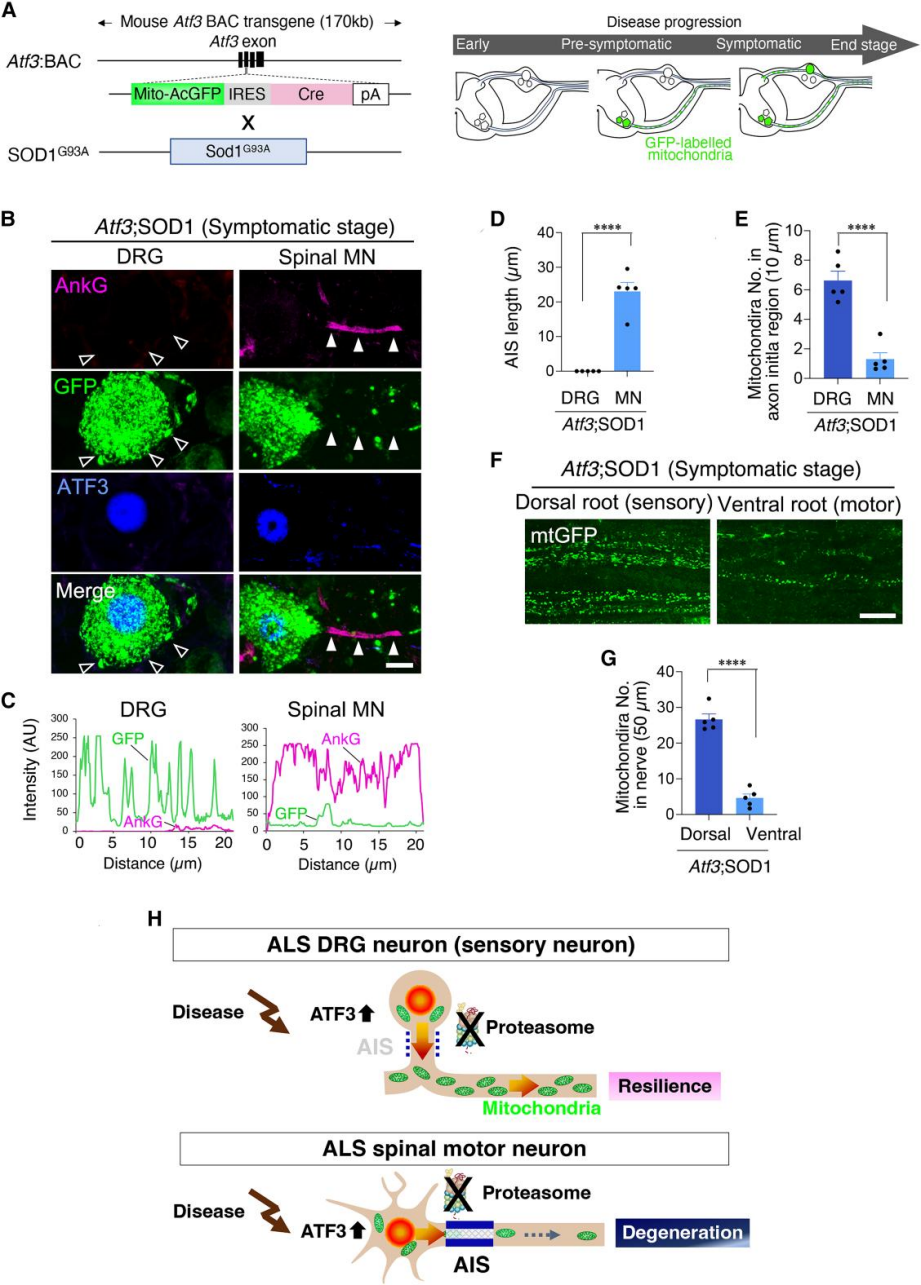
**Pathologically damaged DRG neurons distribute sufficient numbers of mitochondria throughout the axon in ALS mice.** (**A**) Schematic diagram demonstrating a *Atf3*:BAC; SOD1^G93A^ (*Atf3*;SOD1) mouse. The *Atf3*;SOD1 mouse induces the expression of GFP against pathological damage along with disease progression. (**B**) The expression of AnkG, GFP and ATF3 in a DRG neuron and spinal motor neuron of *Atf*3;SOD1 mice at the symptomatic stage. Open arrowheads show the GFP-labelled mitochondria in the absence of AIS, and filled arrowheads indicate few GFP-labelled mitochondria in the AIS. (**C**) Graphs show the GFP and AnkG fluorescent intensity scans corresponding to **B** over a 20 μm line running from the soma to the axon. (**D**) The length of the AIS in DRG and spinal motor neurons of *Atf*3;SOD1 at the symptomatic stage. (**E**) The number of GFP-labelled mitochondria in the beginning of the axon in *Atf3*;SOD1 mice. (**F**) Whole-mount observation of GFP-labelled mitochondria in dorsal root and ventral root of *Atf3*;SOD1 at the symptomatic stage. (**G**) The graph shows the number of axonal mitochondria per 50 µm of sensory dorsal root and motor ventral root of *Atf3*;SOD1 at the symptomatic stage. (**H**) Predicted hypothesis regarding the reason why DRG neurons have a high threshold against pathological damage in ALS. Scale bars = 10 µm in **B** and **F**. Data are shown as the mean ± standard error of the mean. ****P < 0.0001, determined by Student's *t*-test one-way ANOVA followed by Tukey’s *post hoc* analysis, *n* = 5 mice per group. AIS = axon initial segment; ALS = amyotrophic lateral sclerosis; CKO = conditional knockout; DRG = dorsal root ganglion; GFP = green fluorescent protein; MN = motor neuron.

To examine AIS dynamics and mitochondrial behaviour in ALS pathology further, we crossed *Atf3*:BAC Tg mice with SOD1^G93A^ mice to generate *Atf3*;SOD1 mice (Fig. 6A). ALS-damaged neurons are labelled in *Atf3*;SOD1 mice (Fig. 6A and B) because both DRG neurons and spinal motor neurons induce ATF3 in response to the pathological damage that occurs with disease progression in SOD1 mice (Fig. 1F–I). *Atf3*;SOD1 mice had a similar lifespan and similar age-dependent motor neuron death to SOD1 mice, indicating that the *Atf3*:BAC transgene does not affect the disease phenotype of SOD1 mice, which is consistent with our previous observations.^19^ We used *Atf3*:BAC2 Tg mice and not *Atf3*:BAC Tg mice as Cre driver mice to generate the *Rpt3* CKO mice represented in Figs 3–5. *Atf3*:BAC Tg mice and *Atf3*:BAC2 Tg mice are different lines with the same exogenous transgene. Both lines respond to nerve injury in an ATF3-dependent manner. However, the progeny of *Rpt3* CKO mice crossed with *Atf3*:BAC Tg mice had a shorter lifespan, probably because of unexpected expression of Cre recombinase during development. For this technical reason, we used *Atf3*:BAC2 Tg mice to generate *Rpt3* CKO mice. With respect to GFP expression, adult *Atf*3:BAC Tg mice reflected the expression pattern of endogenous ATF3 more precisely than adult *Atf3*:BAC2 Tg mice. We therefore used *Atf3*:BAC Tg mice to generate the *Atf3*;SOD1 mice represented in Fig. 6. The mice were not affected by the expression of Cre recombinase because SOD1 mice do not have loxP sites.

As we found previously,^19^ ALS motor neurons with proteasome dysfunction failed to disassemble the AIS in response to pathological damage, in a similar manner to injury-induced *Rpt3*-deficient motor neurons. We therefore attempted to examine the AIS in pathologically damaged DRG neurons at the symptomatic stage in *Atf3*;SOD1 mice. As shown in Fig. 6B–E, the pathologically damaged DRG neurons in *Atf3*;SOD1 mice did not show an AnkG+ AIS, while GFP-labelled mitochondria were frequently observed in the region. In contrast, pathologically damaged spinal motor neurons in *Atf3*;SOD1 mice retained the AIS, while mitochondria were excluded from it (Fig. 6B–E), as described previously.^19^ Distal axons of DRG neurons in *Atf3*;SOD1 mice had normal numbers of mitochondria (Fig. 6F and G). However, the numbers of axonal mitochondria were severely restricted in the motor axons of *Atf3*;SOD1 mice at the symptomatic stage (Fig. 6F and G).

## Discussion

Protein aggregation causes neuronal dysfunction and neurodegeneration. It is believed that responses to pathological damage contribute to neuronal resilience long before the accumulation of protein aggregates. Sensory DRG neurons seem to have a resilience mechanism that enables them to escape ALS pathological damage. To investigate this issue, we used a nerve injury model in combination with unique injury-induced genetically engineered mice. Using the strategy, we determined that sensory DRG neurons lack the typical AIS structure, which facilitates an increase in damage-induced mitochondrial influx into the axon with or without proteasomes (Fig. 6H).

### Our nerve injury model is useful for exploring early stress-resilience mechanisms during pathological damage

The nerve injury model and our unique mouse system using *Atf3*:BAC Tg mice provide a powerful strategy to examine responses to both pathological and traumatic damage. Using this system, we revealed why sensory DRG neurons are resistant to traumatic and pathological damage under proteasome deficiency, whereas spinal motor neurons degenerate. ATF3 is a well-established marker of nerve injury. Recently accumulated evidence shows that ATF3 expression is induced in neurons, not only after traumatic nerve injury, but also in a variety of stressful conditions, such as demyelination and ALS or because of a disease-causing gene mutation or gene deficiency.^6-9^ Our study also shows that both motor neurons and sensory DRG neurons induce the expression of ATF3 as ALS pathology progresses. It is likely that nerve-injured neurons and diseased neurons share multiple damage responses. In this context, nerve injury is useful as a simple and elegant model to exclude complicating factors of disease and to identify damage responses.

ATF3 acts on both protective and apoptotic pathways. In CNS injury, ATF3 drives a pro-apoptotic pathway in injured neurons.^42-44^ In contrast, injury to the peripheral nervous system induces robust expression of ATF3 for neuronal survival and regeneration. The overexpression of ATF3 or the combination of ATF3 overexpression with dual leucine zipper kinase (DLK) deletion in ALS mice delays degeneration of motor neurons.^45,46^ The function of ATF3 might therefore depend on the cellular context and transcriptional complexes including dimerization partners.^36,47^ Either way, it is certain that ATF3 expression indicates a response to damage and the initiation of an injury-induced transcriptional programme.

### The resistance of damaged DRG neurons to disrupted protein homeostasis

The proteasome system is the main pathway to degrade proteins in cells. It is expected that damaged neurons require rapid protein turnover to shut down cell signalling and to initiate a status of regeneration. Impaired proteostasis has been suggested to be a key factor in neurodegenerative diseases.^19,27,48^ One possibility for the reason why damaged DRG neurons are not affected by proteasome deficiency is that the cellular processes in injured DRG neurons do not require proteasome-dependent proteolysis. However, Kong *et al*.^49^ reported that the proteasome contributes to AMPK degradation, which promotes axonal regeneration of DRG neurons. The membrane proteasome is also reported to have a critical role in DRG neurons.^50^ The application of a proteasome inhibitor to DRG neurons affects cytoskeletal axonal transport and neuropathic pain.^51-54^ These findings indicate that the proteasome plays a crucial role in DRG neurons in response to injury. Alternatively, damaged DRG neurons might have an unknown stress-resilience mechanism to compensate for proteasome deficiency. The lack of the AIS in DRG neurons might provide a fascinating explanation for the resistance of sensory DRG neurons to pathological damage. However, we do not exclude other possibilities, such as the autophagy system compensating for proteasome deficiency in injured DRG neurons.

### Absence of the AIS is advantageous for DRG neurons to resist damage under proteasome dysfunction

Healthy mitochondria are supplied to axons from soma to satisfy the axonal energy demand. Proper regulation of mitochondrial content is therefore important for axonal integrity. Previous studies using genetically engineered mice and 3D scanning electron microscopy with a focused ion beam revealed a unique and exclusive relationship between the AIS and mitochondria in the initial part of the axon in motor neurons.^19,41^ Normally, mitochondria are rarely observed in the region of the AIS, but they are abundant at the site of proteasome-dependent AIS disassembly, and their number increases in the axon in response to nerve injury. Injured motor neurons that are proteasome deficient fail to dismantle the AIS. The knockdown of AnkG in proteasome-deficient injured motor neurons increases the number of mitochondria in the beginning of the axon and partly rescues neuronal death.^19^ This exclusive relationship between mitochondria and the AIS is also maintained in cortical neurons.^55,56^ In axons, the majority of mitochondria are stationary, and the remainder are motile. The AIS structure seems to exclude stationary mitochondria and to restrict the entry of motile mitochondria into axons. Therefore, the transient proteasome-mediated dismantling of the AIS upon motor axon injury is critical for the increased mitochondrial influx into axons. We initially hypothesized that DRG neurons have a compensatory mechanism beyond AIS disassembly in response to damage with proteasome dysfunction. Intriguingly, we did not detect the typical AnkG+ AIS, even in normal DRG neurons, but we did identify the AIS in motor, cortical and hippocampal neurons and in retinal ganglion cells. This was supported by data showing that the initial part of the axon in DRG neurons, where mitochondria were abundantly localized, coils up like a snake. Given previous studies that show a mutually exclusive relationship between the AIS and mitochondria, we conclude that DRG neurons might lack the typical AnkG+ AIS structure.

Existence of the AIS in DRG neurons is controversial both *in vitro* and *in vivo*. Conflicting reports have described the presence and absence of the AIS structure in DRG neurons.^24^ Nascimento *et al*.^25,26^ showed the AIS to be present in DRG neurons *in vivo* and that deletion of AnkG, the AIS master organizer, in DRG neurons relieves pain behaviour in sensory neuron-specific AnkG deletion mice. We are not sure whether deletion of AnkG affects the AIS specifically or if it also affects the node of Ranvier, because it is composed of the same protein components as the AIS, including AnkG. The Rasband group performed experiments using sensory neuron-specific AnkG-deficient mice. Sensory nerve regeneration was not affected in these mice.^57^ However, they showed that motor neuron-specific AnkG-deficient mice had delayed functional recovery after nerve injury, probably because of the impaired reappearance of the AIS. These data indicate that DRG neurons lack the AIS and do not need to reassemble the AIS for regeneration. We do not rule out the possibility of an *in vivo* AIS-like compartment in DRG neurons or of another region, such as the pre-axonal exclusion zone, that can generate an action potential.^58^ However, the lack of a typical AnkG+ AIS can explain why DRG neurons in ALS have such high resilience against damage and increased mitochondrial content in damaged axons with proteasome deficiency.

## Conclusion

Overall, this study demonstrates an important mechanism for sensory neuron resilience in pathological conditions, which includes proteasome dysfunction in the early stage of ALS. Understanding responses to disease-induced damage can provide new insight for early diagnosis and clinical intervention. However, there might be additional unknown proteasome-dependent or -independent mechanisms that affect resilience against pathological damage before protein aggregation becomes apparent in ALS. Moreover, it is not clear why DRG neurons lack the typical AIS in normal conditions. Further study and technical advances are required to resolve these issues.

## Supplementary Material

awaf182_Supplementary_Data

## Data Availability

The authors confirm that the data supporting the findings of this study are available within the article and its Supplementary material.
