# Supplementary material for: Absence of the axon initial segment in sensory neuron enhances resistance to amyotrophic lateral sclerosis
Source: Brain. 2025 Jul 7;148(11):4030–44. doi: 10.1093/brain/awaf182 (PMC12588706; doi:10.1093/brain/awaf182)
Supplement: awaf182_Supplementary_Data [file awaf182_supplementary_data.zip › BRAIN-2024-03209_Author_contributions.pdf]

Author contributions

N.T.T., S.K.-S., and Hi.K. conceived and designed the project. N.T.T. and S.K.-S. performed experiments. S.K.-S. generated mice. Ha.K and K.W. performed immunostaining using AIS marker antibodies. M.T., M.A., and T.N. acquired and analyzed 3D images. Y.T. and R.T. provided the *Rpt3*$^{f/f}$ mice. Y.I. and M.K. provided SOD1^{G93A} mice. N.T.T., S.K.-S., and Hi.K. wrote the paper with contributions from all authors.
