# Supplementary material for: Absence of the axon initial segment in sensory neuron enhances resistance to amyotrophic lateral sclerosis
Source: Brain. 2025 Jul 7;148(11):4030–44. doi: 10.1093/brain/awaf182 (PMC12588706; doi:10.1093/brain/awaf182)
Supplement: awaf182_Supplementary_Data [file awaf182_supplementary_data.zip › BRAIN-2024-03209_Supplementary_figures_and_legends.pdf]

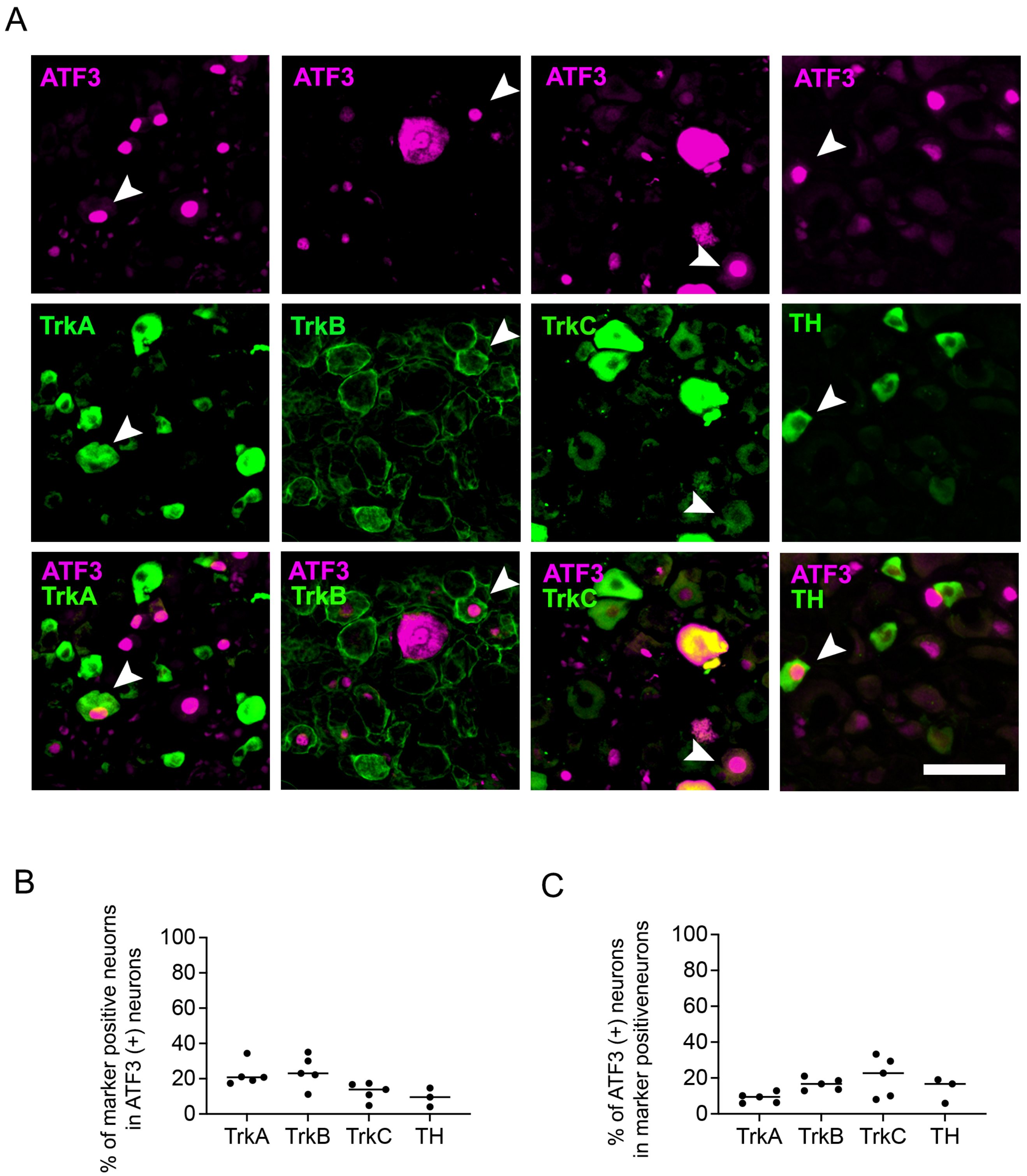

Supplementary Figure 1_Tra et al.

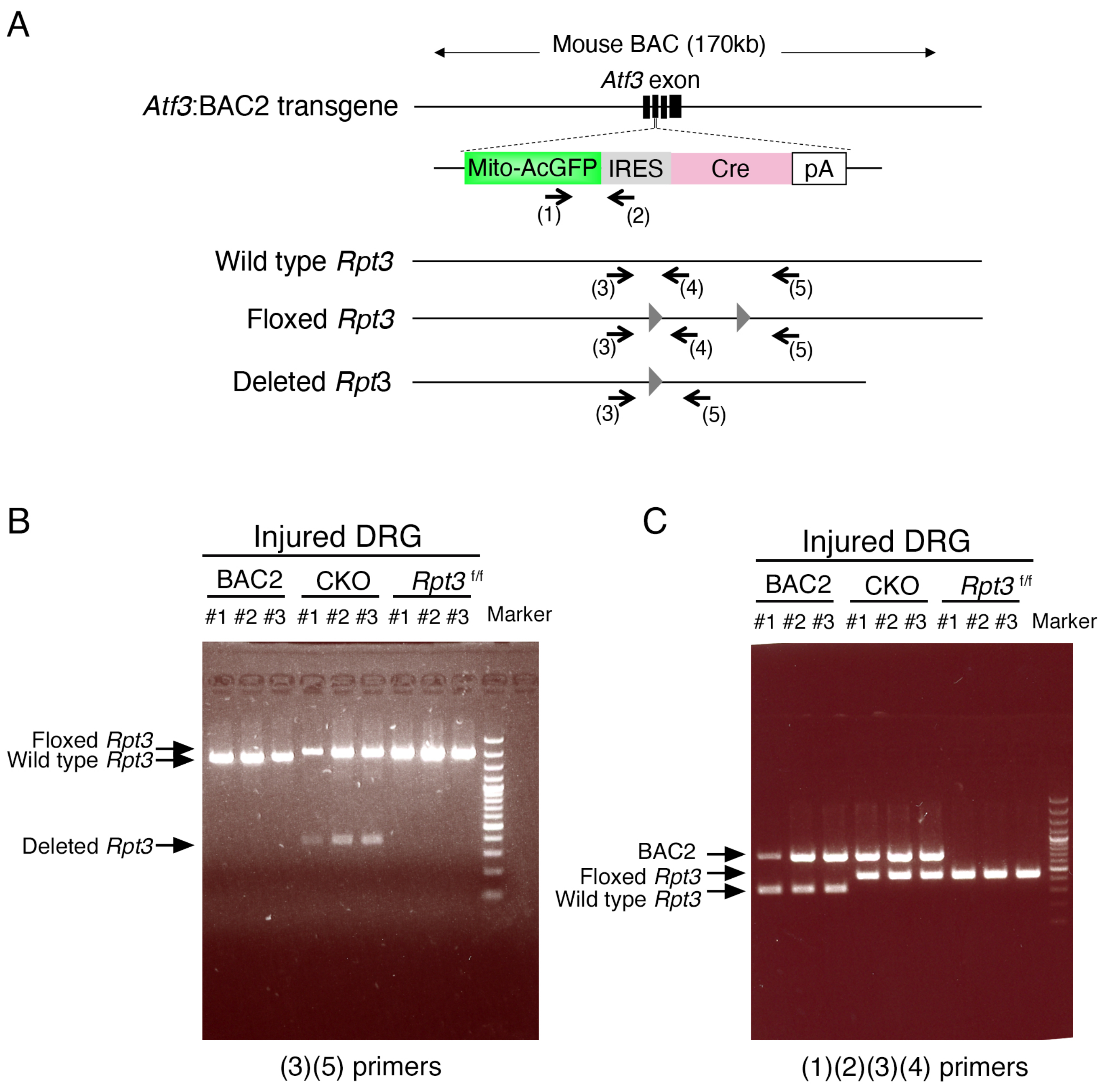

Supplementary Figure 2_Tra et al.

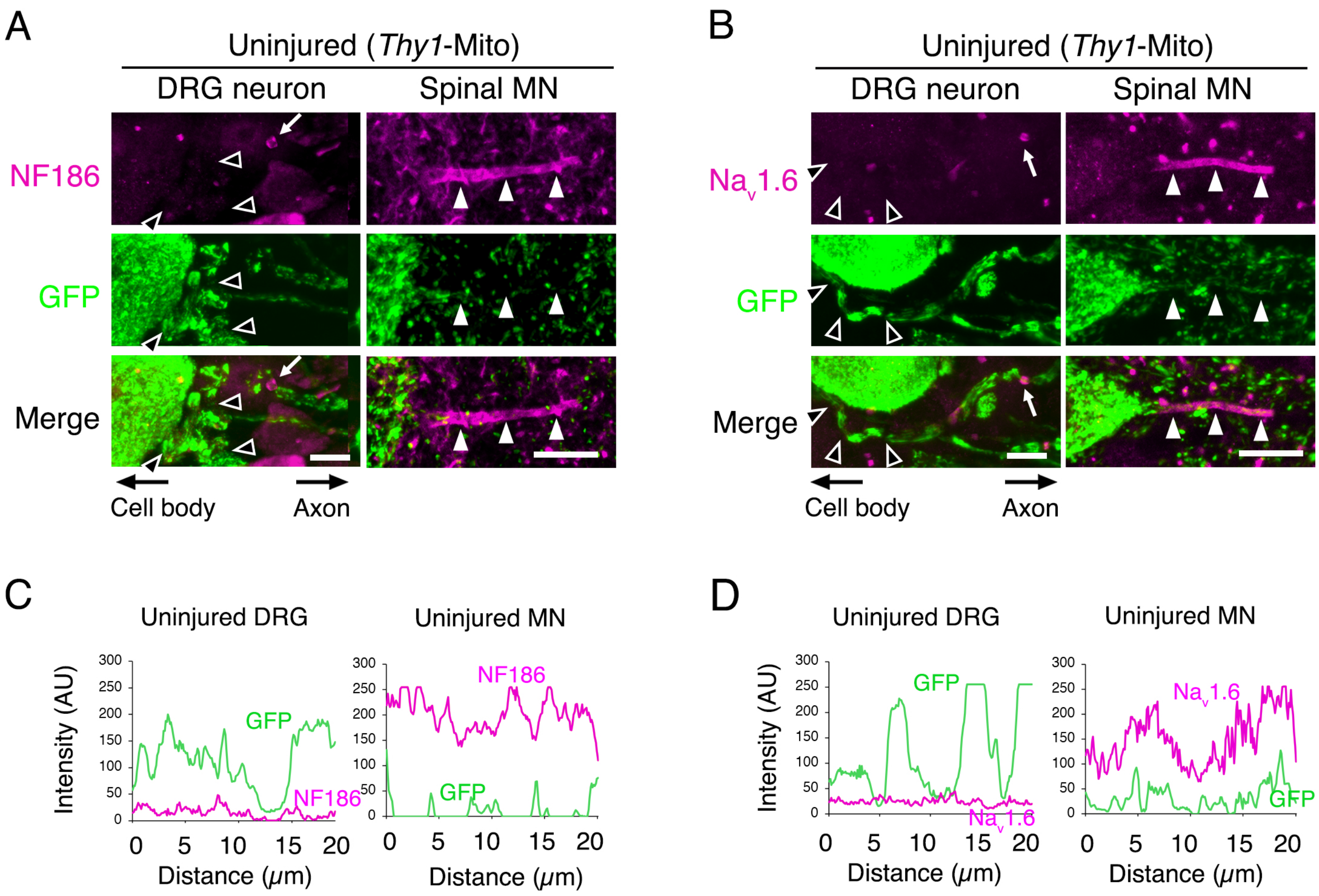

Supplementary Figure 3_Tra et al.

Figure S1. Identification of ATF3-positive DRG neurons in end-stage SOD1^{G93A} ALS mice

(**A**) Immunostaining of DRGs in SOD1^{G93A} mice for ATF3 and for TrkA, TrkB, TrkC, and TH, which are representative markers of different cell subtypes. Arrowheads show the co-localization of each marker with ATF3. (**B**) Graph shows the percentage of marker-positive neurons in all ATF3-positive neurons. (**C**) Graph shows the percentage of ATF3-positive neurons in marker-positive neurons. Data are shown as the mean ± s.e.m., determined by one-way ANOVA followed by *Tukey* post-hoc test, n = 3–5 mice per group. Scale bar, 50 µm in (A).

Figure S2. Genotyping *Atf3*:BAC2 Tg and *Rpt3* CKO mice

(**A**) Diagram of the *Atf3*:BAC2 transgene and *Rpt3* gene. Arrows indicate the location of the genotyping primers (1)–(5) for *Atf3*:BAC2 Tg and *Rpt3* CKO mice. The gray triangles show loxP sites. (1)-(5) primers correspond to *Atf3*:BAC2-F, *Atf3*:BAC2-R, *Rpt3*-F, *Rpt3*-R and *Rpt3*-delta primers respectively. (**B**) Representative genotyping results using primers (3) and (5) to detect the deleted *Rpt3* gene in DRGs at 5 days after sciatic nerve injury. Mouse samples #1–#3 were collected from *Atf3*:BAC2 Tg, *Rpt3* CKO, and *Rpt3*$^{f/f}$ mice, respectively. (**C**) Representative genotyping using primers (1)–(4). Marker: GeneRuler 100 bp Plus DNA Ladder (SM0321, Thermo Fisher Scientific).

Figure S3. Neurofascin 186 and Nav1.6 in sensory DRG neurons and spinal motor neurons

(**A** and **B**) Neurofascin 186 in (**A**), Nav1.6 in (**B**) and GFP in (**A** and **B**) in the initial part of the axon from DRG neurons and spinal motor neurons of *Thy1*-Mito mice under normal conditions. Open arrowheads indicate the constitutive localization of GFP-labeled mitochondria in the beginning of the axon while closed arrowheads indicate few mitochondria in the AnkG-positive AIS region. Arrows indicate the node of Ranvier. (**C** and **D**) Graphs show the GFP and Neurofascin 186 (**C**) and the GFP and Nav1.6 (**D**) fluorescence intensity scans corresponding to (**A**) and (**B**), respectively, over a 20 µm line running from the soma to the axon. Scale bars, 10 µm in (**A** and **B**).
