# Supplementary material for: Absence of the axon initial segment in sensory neuron enhances resistance to amyotrophic lateral sclerosis
Source: Brain. 2025 Jul 7;148(11):4030–44. doi: 10.1093/brain/awaf182 (PMC12588706; doi:10.1093/brain/awaf182)
Supplement: awaf182_Supplementary_Data [file awaf182_supplementary_data.zip › BRAIN-2024-03209_Supplementary_Table_1.pdf]

Supplementary Table 1 Antibody list

Antibodies	Source	Catalog number or identifier
Rabbit anti-GFP polyclonal (1:1,000)	MBL	Cat# 598, RRID:AB_591819
Rat anti-GFP monoclonal (1:1,000)	Nacalai Tesque	Cat# 04404-84, RRID:AB_10013361
Rabbit anti-Ankirin-G polyclonal (1:1000)	Frontier Institute	Cat# AnkG-Rb, RRID:AB_2571661
Mouse anti-Ankyrin-G Monoclonal (1:1000)	NeuroMab	Cat# 75-146, RRID:AB_10673030
Rabbit anti-RPT3 polyclonal (1:500)	Proteintech	Cat# 11389-1-AP, RRID:AB_2300373
Guinea pig anti-p62 polyclonal (1:500)	Progen	Cat# GP62-C, RRID:AB_2687531
Mouse anti-NeuN monoclonal (1:1,000)	Millipore	Cat# MAB377, RRID:AB_2298772
Rabbit anti-ATF-3 (C-19) polyclonal (1:1,000)	Santa Cruz Biotechnology	Cat# sc-188, RRID:AB_2258513
Rabbit anti-ATF3 monoclonal (1:1,000)	Abcam	Cat# ab207434, RRID:AB_2734728
Rabbit anti-Tuj1 polyclonal (1:5000)	Abcam	Cat# ab18207, RRID:AB_444319
Goat anti-TrkA polyclonal (1:500)	R and D Systems	Cat# AF1056, RRID:AB_2283049
Goat anti-TrkB polyclonal (1:500)	R and D Systems	Cat# AF1494, RRID:AB_2155264
Goat anti-TrkC polyclonal (1:100)	R and D Systems	Cat# AF1404, RRID:AB_2155412
Sheep anti-Tyroshine Hydroxylase (TH) (1:200)	Novus	Cat# NB300-110, RRID:AB_ 350438
Rabbit anti-Iba1 polyclonal (1:1,000)	Wako	Cat# 019-19741, RRID:AB_839504
Rabbit anti-Nav1.6 polyclonal (1:500)	Alomone lab	Cat# ASC-009, RRID:AB_2040202
Rabbit anti-Neurofascin 186 monoclonal (1:1000)	Cell signaling	Cat# 15034, RRID:AB_2773024
Alexa Donkey anti-rat 488 (1:1000)	Thermo Fisher Scientific	Cat# A-21208, RRID:AB_2535794
Alexa Donkey anti-mouse 488 (1:1000)	Thermo Fisher Scientific	Cat# A-21202, RRID:AB_141607
Alexa Donkey anti-goat 488(1:1000)	Thermo Fisher Scientific	Cat# A-11055, RRID:AB_253410
Alexa Donkey anti-sheep 488(1:1000)	Thermo Fisher Scientific	Cat# A-11015, RRID:AB_2534082
Alexa Donkey anti-rabbit 594 (1:1000)	Thermo Fisher Scientific	Cat# A-21207, RRID:AB_14163
Alexa Goat anti-guina pig 594 (1:1000)	Thermo Fisher Scientific	Cat# A-11076, RRID:AB_2534120
Alexa Donkey anti-goat 647 (1:1000)	Thermo Fisher Scientific	Cat# A-21447, RRID:AB_2535864
Alexa Donkey anti-rabbit 647 (1:1000)	Thermo Fisher Scientific	Cat# A-31573, RRID:AB_2536183
4',6-diamidino-2-zhenylindole (DAPI)	Dojindo	D523
